# Structural characterisation of nucleotide sugar short‐chain dehydrogenases/reductases from the thermophilic pseudomurein‐containing methanogen *Methanothermobacter thermautotrophicus* ΔH

**DOI:** 10.1111/febs.70248

**Published:** 2025-09-03

**Authors:** Vincenzo Carbone, Linley R. Schofield, Patrick J. B. Edwards, Andrew J. Sutherland‐Smith, Ron S. Ronimus

**Affiliations:** ^1^ AgResearch Ltd. Grasslands Palmerston North New Zealand; ^2^ School of Food Technology and Natural Sciences Massey University Palmerston North New Zealand

**Keywords:** cell wall, dehydratase, epimerase, Methanothermobacter, pseudomurein, SDR

## Abstract

Epimerases and dehydratases are widely studied members of the extended short‐chain dehydrogenase/reductase (SDR) enzyme superfamily and are important in nucleotide sugar conversion and diversification, for example, the interconversion of uridine diphosphate (UDP)‐linked glucose and galactose. *Methanothermobacter thermautotrophicus* contains a cluster of genes, the annotations of which indicate involvement in glycan biosynthesis such as that of cell walls or capsular polysaccharides. In particular, genes encoding UDP‐glucose 4‐epimerase related protein (*Mth375*), UDP‐glucose 4‐epimerase homologue (*Mth380*) and dTDP‐glucose 4,6‐dehydratase related protein (*Mth373*) may be involved in the biosynthesis of an unusual aminosugar in pseudomurein. In this paper, we present the structures of Mth375, an archaeal sugar epimerase/dehydratase protein (WbmF) determined to a resolution of 2.0 Å. The structure contains an N‐terminal Rossmann‐fold domain with bound nicotinamide adenine dinucleotide hydride (NADH) and a C‐terminal catalytic domain with bound UDP. We also present the structure for Mth373 co‐crystallised with uridine‐5′‐diphosphate‐xylopyranose to a resolution of 1.96 Å as a NAD^+^‐dependent oxidative decarboxylase (UDP‐xylose synthase; EC4.1.1.35). Molecular modelling has also allowed for the identification of Mth380 as a UDP‐*N*‐acetylglucosamine 4‐epimerase (WbpP; EC5.1.3.7), Mth631 as a UDP‐glucose 4‐epimerase (GalE; EC5.1.3.2) and Mth1789 as a classical dTDP‐d‐glucose 4,6‐dehydratase (EC4.2.1.46). The UDP–sugar specificity of each archaeal nucleotide sugar short‐chain dehydrogenase/reductase (NS‐SDR) was elucidated via sequence, molecular modelling and structural analyses. Overall, these structures potentially shed light on the formation of the glycan portion of pseudomurein and capsular polysaccharide in Archaea.

## Graphical abstract

Nucleotide sugar short‐chain dehydrogenases/reductases (NS‐SDRs) are involved in pseudomurein and capsular polysaccharide formation in methanogenic Archaea. *Methanothermobacter thermautotrophicus* possesses several NS‐SDRs labelled Mth375, Mth380, Mth373, Mth631 and Mth1789. Utilising X‐ray crystallography, molecular modelling and phylogenetics we shed light on the NS‐SDRs of *M. thermautotrophicus* and their potential enzyme activities.

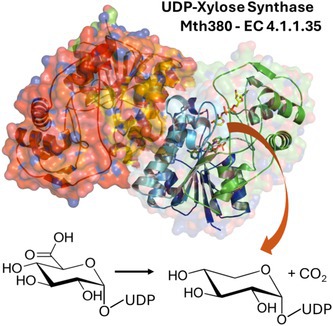

**Abbreviations**
AMP: adenosine 5′‐monophosphate
CEP1: carbohydrate epimerase family 1
CME: S,S‐(2‐hydroxyethyl)thiocysteine
Da: Dalton
DAU: 2′deoxy‐thymidine‐5′‐diphospho‐ α‐d‐glucose
DNA: deoxyribonucleic acid
dTDP: thymidine diphosphate
dTGD: dTDP‐glucose 4,6‐dehydratase
DTT: dithiothreitol
EPZ: UDP‐*N*‐acetyl‐α‐d‐muramic acid
EX‐L: extended loops
GDP: guanosine diphosphate
Glc: glucose
GlcN: glucosamine
GlcNAc: *N*‐acetylglucosamine
GOL: glycerol
HEPES: 2‐[4‐(2‐hydroxyethyl)piperazin‐1‐yl]ethanesulfonic acid
HP7: UDP‐*N*‐acetyl‐d‐glucosaminuronate
LPS: lipopolysaccharide
MOPS: 3‐(*N*‐morpholino)propanesulfonic acid
Mth: *Methanothermobacter thermautotrophicus* ΔH
NAcTalNA: *N*‐acetyltalosaminuronic acid
NADH: nicotinamide adenine dinucleotide hydride
NAT: *N*‐acetyl‐l‐talosaminuronic
NCBI: National Center for Biotechnology Information
NS‐SDR: nucleotide sugar short‐chain dehydrogenases/reductases
PDB: Protein Data Bank
PEG: polyethylene glycol
PMSF: phenylmethylsulfonyl fluoride
RCSB: Research Collaboratory for Structural Bioinformatics
RMSD: root mean square deviation
SDR: short‐chain dehydrogenase/reductase
SDS/PAGE: sodium dodecyl sulfate polyacrylamide gel electrophoresis
TCEP: tris (2‐carboxyethyl) phosphine
TM: transmembrane)
Tris: tris(hydroxymethyl)aminomethane
TYD: thymidine‐5′‐diphosphate
UAXS: UDP‐d‐apiose/UDP‐d‐xylose synthase
UD1: UDP‐*N*‐acetylglucosamine
UDP: uridine‐5′‐diphosphate
UDP‐GlcNAc: UDP‐*N*‐acetylglucosamine
UDX: UDP‐xylopyranose
UGA: UDP‐glucuronic acid
UPG: UDP‐glucose
UXS: UDP‐α‐d‐xylose synthase

## Introduction

**Fig. 1:**
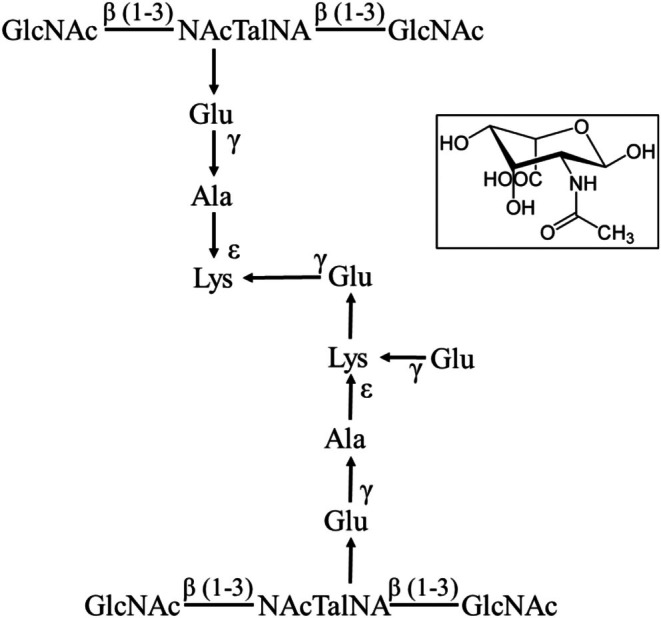
Methanogen pseudomurein. Methanogen pseudomurein contains amino sugars in the glycan backbone linked through β (1–3) bonds, the unusual amino sugar NAcTalNA, L‐amino acids and several unusual isopeptide bonds. Inset: The structure of NAcTalNA. The figure was adapted and modified from Schofield *et al*. [77]. GlcNAc, *N*‐acetylglucosamine; NAcTalNA, *N*‐acetyltalosaminuronic acid.

Cell walls are important for the survival of prokaryotes by enabling the preservation of cell shape from osmotic stresses [1, 2, 3, 4]. Almost all bacteria contain peptidoglycan (murein) in their cell walls, the biosynthesis of which has been extensively investigated [5, 6, 7, 8], while archaea possess an array of cell walls none of which are closely related to peptidoglycan [2, 9]. These include methanochondroitin, sulfated heteropolysaccharides, S‐layers, proteinaceous sheaths, glutaminylglycans, halomucin, polysaccharide‐based glycocalyces or pseudomurein, depending on their phylogenetic affiliation [2, 9, 10, 11, 12, 13, 14]. The latter cell wall type, pseudomurein, is found in the orders Methanobacteriales and Methanopyrales and at a very basic component level, is analogous to peptidoglycan in that it contains both a glycan backbone and a peptide cross‐link [15, 16, 17]. However, the amino sugars in the glycan backbone are linked through β(1–3) bonds, the peptide cross‐link only contains L‐amino acids and the pentapeptide incorporates several isopeptide bonds [10, 16, 17]. In addition, pseudomurein universally contains an unusual amino sugar moiety, *N*‐acetyl‐l‐talosaminuronic (NAT) acid which typically alternates with either *N*‐acetylglucosamine or *N*‐acetylgalactosamine residues (Fig. 1) [15, 16, 17, 18, 19]. Neutral nonacetylated sugar residues glucose (Glc) and glucosamine (GlcN) can also be present. The presence of *N*‐acetylglucosamine or *N*‐acetylgalactosamine residues varies dependent on species [10]; for *Methanothermobacter thermautotrophicus* ΔH, *N*‐acetylglucosamine residues predominate [10]. The overall differences in chemistry and biosynthesis between pseudomurein and peptidoglycan are large enough that it has been hypothesised that the two cell wall types are likely to have evolved independently [2, 16, 17], though the bacterial Mur and archaeal pMur peptide ligases share a common evolutionary history [20, 21].

The biosynthesis of the unusual *N*‐acetyl‐l‐talosaminuronic acid in pseudomurein is hypothesised to be achieved by epimerisation and oxidation of UDP‐*N*‐acetylgalactosamine. Methanobacterial and *Methanopyrus kandleri* genomes contain easily identifiable orthologues of bacterial GlmS (glucosamine‐fructose‐6‐phosphate aminotransferase; EC2.6.1.16), GlmM (phosphoglucosamine mutase; EC5.4.2.10) and the bifunctional GlmU (bifunctional UDP‐*N*‐acetylglucosamine pyrophosphorylase/glucosamine‐1‐phosphate *N*‐acetyltransferase; EC2.7.7.23 and EC2.3.1.157). These enzymes have the capability to produce UDP‐*N*‐acetylglucosamine, an amino sugar residue in the glycan backbone of most pseudomurein‐containing methanogens [10, 11, 16, 17, 22, 23]. In addition, most of these genomes also contain at least one annotated UDP‐*N*‐acetylglucosamine 2‐epimerase (WecB; EC5.1.3.14) and all contain at least one UDP‐glucose 4‐epimerase (GalE; EC5.1.3.2 [22]), although both of these epimerase types have not yet been functionally characterised from any methanobacterium or *Methanopyrus kandleri* strain [24, 25]. In recent efforts, we have structurally characterised a WbpP (UDP‐*N*‐acetylglucosamine 4‐epimerase; EC5.1.3.7) from *Methanobrevibacter ruminantium* [26].

Van Overtveldt *et al*. [27] reviewed carbohydrate epimerases and classified them based on reaction mechanisms and structure. The CEP1 family (carbohydrate epimerase family 1) includes both UDP‐glucose 4‐epimerases (EC5.1.3.2) and GDP‐mannose 3,5‐epimerases (EC5.1.3.18) with the same reaction mechanism [27]. This suggests that *M*. *thermautotrophicus* UDP‐glucose 4‐epimerase orthologues could potentially encode enzymes that are capable of 3,5‐epimerisation. Interestingly, protein sequence searches indicate that *M. thermautotrophicus* ΔH potentially possesses several enzymes with UDP‐glucose 4‐epimerase activity (Mth373, Mth375, Mth380, Mth631 and Mth1789) [22, 28]. These enzymes are found in a large gene cluster in *M. thermautotrophicus* suggestive of a role in either cell wall biosynthesis or capsular polysaccharide formation [20, 21, 28], and with Mth375 and Mth380 annotated as UDP‐glucose 4‐epimerase‐related enzymes, and Mth373 annotated as a dTDP‐glucose 4,6‐dehydratase (dTGD) related protein. Additional enzymes of note within this gene cluster include Mth370 a LPS biosynthesis RfbU‐related protein, Mth371 a glycosyltransferase RgtA/B/C/D‐like domain‐containing protein, Mth374 a dolichyl‐phosphate mannose synthase‐related protein, Mth376 a glycosyl transferase‐related protein, Mth377 a dolichyl‐phosphate mannose synthase‐related protein, Mth378 a lysylphosphatidylglycerol synthase TM region and Mth379 an *O*‐antigen transporter‐related enzyme.

To gain a better understanding of *N*‐acetyl‐l‐talosaminuronic acid synthesis and more generally aminosugar conversions in pseudomurein‐containing methanogens, we have chosen to investigate NS‐SDRs from the model species *Methanothermobacter thermautotrophicus* ΔH for structural studies. In this paper, we present two structures of Mth375, an archaeal sugar epimerase/dehydratase (WbmF; with UDP bound) and two structures of Mth373, a NAD^+^‐dependent oxidative UDP‐glucuronic acid decarboxylase (EC 4.1.1.35; with UDX bound). The enzyme structures revealed that Mth375 has a similar fold and active site to bacterial GalE 4‐epimerases (EC5.1.3.2), UDP‐glucuronate decarboxylases (EC4.1.1.35), WbpP, and WbgU (EC5.1.3.7) enzymes, while Mth373 shares conserved protein domains with the NAD^+^‐dependent epimerase/dehydratase family of proteins, including WcaG [29, 30] placing both enzymes within the SDR superfamily. Molecular modelling and sequence alignment enabled the functional prediction of Mth380 as a WbpP (EC5.1.3.7), Mth631 as another GalE 4‐epimerase (EC5.1.3.2) and Mth1789 as a classical dTGD (EC4.2.1.46).

## Results and discussion

### Crystal structure of Mth375

**Fig. 2:**
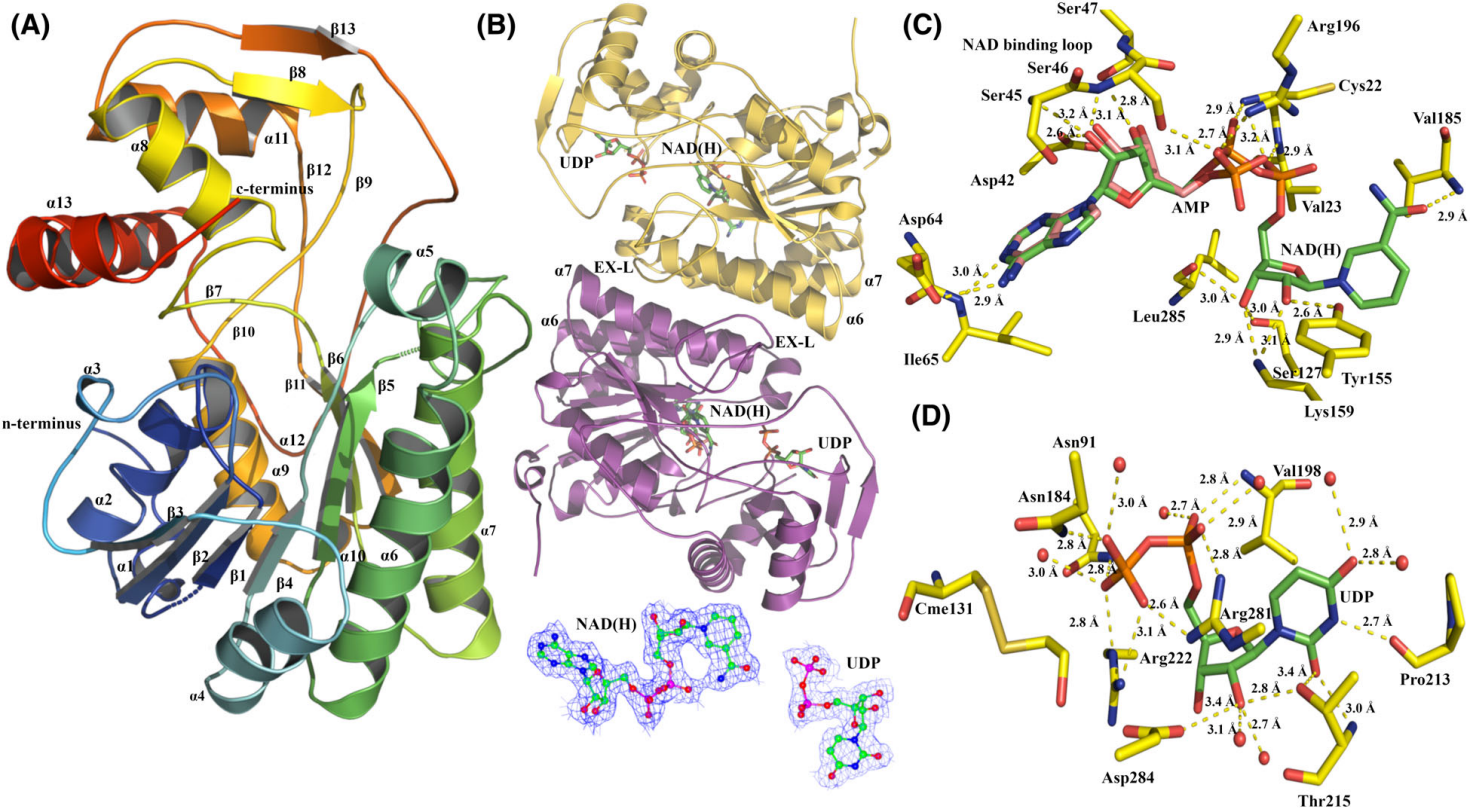
Structures of *M. thermautotrophicus* ΔH Mth375. (A) A ribbon representation of Mth375 (WbmF; nucleotide sugar epimerase/dehydratase) with secondary structural elements labelled. The structure is colour‐ramped from the N terminus (blue) to C terminus (red). (B) Dimeric Mth375_NU with bound molecules, helices α_6_ and α_7_ and the extended loop labelled (EX‐L). Immediately below depicts the 2Fo‐Fc electron density maps of the refined NADH and UDP molecules. (C) The coenzyme‐binding domain of Mth375_NU depicting NAD(H) (carbons in green) and the superimposed AMP molecule bound to Mth375_AU (carbons in pink). (D) The Mth375_NU active site domain with bound UDP (carbons are coloured green). Relevant residues and water molecules are shown (by atom; Mth375 carbons are in yellow). All polar contacts and distances are indicated with dashed lines. All figures were prepared using PyMOL version 2.5.8.

Mth375 expressed with high yield in culture and did not require further purification after nickel‐affinity chromatography, eluting as a single peak with SDS/PAGE analysis showing a purity in excess of 95%, appropriate for protein crystallisation. Mth375_AU crystallised in the trigonal space group *H 32* with unit cell parameters *a* = 113.8 Å, *b* = 113.8 Å, *c* = 239.9 Å, α = 90.0°, β = 90.0° and γ = 120.0°. There was a single monomer in the asymmetric unit (Fig. 2A) with a solvent content estimated to occupy 65% of the unit cell volume and a Matthews coefficient of 3.51 Å^3^·Da^−1^. Mth375_NU crystallised in the hexagonal space group *P 6*_*3*_ *2 2* with unit cell parameters *a* = 89.3 Å, *b* = 89.3 Å, *c* = 174.7 Å, α = 90.0°, β = 90.0° and γ = 120.0° and contained one monomer in the asymmetric unit, with a solvent content occupying 47% of the unit cell volume and a Matthews coefficient of 2.32 Å^3^·Da^−1^ (see Table 1 for all refinement and geometric statistics). After structure determination and refinement, the electron density for both Mth375 complexes was consistent throughout the structures and was unbroken for the main chain of 332 residues, but with a few instances of side chain disorder at the N terminus. Both crystal structures present a single modified active site cysteine (Cys131), while Mth375_NU has a second modified cysteine (Cys13) derivatised by β‐mercaptoethanol present in the storage buffer to form *S*,*S*‐(2‐hydroxyethyl)thiocysteine (CME). In Mth375_AU a single molecule of adenosine 5′‐monophosphate (AMP) was detected in the N‐terminal coenzyme‐binding domain and a single molecule of UDP in the Mth375 active site. The presence of AMP was a consequence of crystallisation with Silver Bullets Bio screen condition containing 0.20% (w/v) of AMP sodium salt. Mth375_NU contained a single NADH molecule, and like Mth375_AU, a single molecule of UDP was present in the active site of the enzyme. *N*‐acetyl‐d‐glucosamine added to the mother liquor prior to freezing Mth375_NU crystals failed to bind in the crystal structure. For both complexes, the UDP molecules superimpose in an identical manner as do the adenosine moieties of AMP and NADH.

**Table 1:** Data collection and refinement statistics.

	*Mth375_AU* *AMP & UDP* *Mth375*	*Mth375_NU* *NAD & UDP* *Mth375*	*Mth373_NU* *NAD & UDP* *Mth373*	*Mth373_NX* *NAD & UDX* *Mth373*
PDB code	6PMH	6PNL	8W3U	9AR1
Space group	H 3 2	P 63 2 2	P 2 2 21	H 3 2
Unit cell parameters a, b, c (Å) α, β, γ (^o^)	113.8, 113.8, 239.9 90.0, 90.0, 120.0	89.3, 89.3, 174.7 90.0, 90.0, 120.0	54.5, 81.4, 152.4 90.0, 90.0, 90.0	139.3, 139.3, 226.9 90.0, 90.0, 120.0
Wavelength (Å)	0.91170	0.95364	0.95370	0.95368
Temperature (K)	100	100	100	100
Resolution Range (Å)	48.27–2.30	46.5–2.01	45.27–2.00	44.71–1.96
No. of observed ref.^a^	298 565 (29548)	540 821 (36612)	672 781 (29403)	1 186 053 (60087)
No. of unique ref.^a^	26 918 (2607)	28 060 (1958)	48 712 (2617)	59 615 (3405)
*R*_sym_ ^a^	0.176 (0.763)	0.182 (0.932)	0.192 (0.768)	0.162 (0.615)
*R*_pim_ ^a^	0.055 (0.237)	0.043 (0.216)	0.076 (0.304)	0.037 (0.145)
Completeness (%)^a^	100.0 (100.0)	99.7 (96.3)	99.8 (98.0)	98.6 (80.6)
Multiplicity^a^	11.1 (11.3)	19.3 (18.7)	7.3 (7.2)	19.9 (17.6)
I/σ(I)^a^	15.1 (3.7)	16.1 (4.6)	9.4 (2.7)	16.3 (5.3)
CC(1/2)	0.996 (0.905)	0.996 (0.867)	0.994 (0.796)	0.996 (0.951)
Refinement statistics				
Resolution range (Å)	91.17–2.3	87.35–2.01	81.38–1.99	106.5–1.97
All reflections used	26 926	28 075	46 881	60 315
Size R free set (%)	5	5	5	5
All reflections (R free)	1380	1382	2290	2999
R factor (%)	14.71	16.19	16.35	17.24
R free (%)	17.76	18.95	20.39	20.51
Matthews coefficient (Å^3^·Da^−1^)	3.51	2.32	2.29	3.03
Solvent content (%)	64.7	46.5	46.3	59.4
RMSD Bond Length (Å)	0.012	0.0083	0.011	0.11
RMSD Bond Angle (^o^)	1.61	1.40	1.53	1.64
Ramachandran plot				
Residues in favoured regions (%)	94.1	93.1	91.1	91.1
Residues in allowed regions (%)	5.9	6.9	8.9	8.9
Average B factors (Å^2^)				
Protein	25.6	22.2	16.5	27.8
UDP	22.5	21.9	14.0	39.2
AMP	17.1	–	–	17.9
NAD	–	16.2	12.3	22.2
UDX	–	–	–	
Ethylene glycol	–	42.7	27.5	38.2
Water	31.3	31.3	23.6	34.7
SO_4_^2−^	–	65.2	–	–
Glycerol	35.2	–	–	38.9
Cl^−^	60.4	–	30.9	–
PO_4_^3−^	28.3	–	–	–
Mg^2+^	–	–	21.6	–

^a^ Data in the highest resolution shell are given in parentheses.

The Mth375 monomer contains two distinct structural domains and common sequence characteristics that are widespread amongst the SDR enzyme superfamily (Fig. 2A,B) [31, 32]. The domains are an N‐terminal nucleotide‐binding domain (residues 1–183) predominantly formed by a modified Rossmann‐fold motif which in Mth375 binds NADH and a C‐terminal active site domain (residues 184–332) that binds the UDP‐sugar substrate. This fold is common amongst archaeal, bacterial and eukaryotic 4‐epimerases [24, 26, 33], a family of enzymes denoted either as GalE (EC 5.1.3.2; UDP‐glucose/galactose 4‐epimerases), WbpP or WbgU (EC 5.1.3.7; UDP‐*N*‐acetylglucosamine/galactosamine 4‐epimerases). The N‐terminal coenzyme‐binding domain incorporates a series of seven parallel β‐strands (residues 14–18 β_1_, 37–42 β_2_, 57–63 β_3_, 80–85 β_4_, 123–130 β_5_, 176–183 β_6_ and the C‐terminal residues 246–250 β_11_) and ten α‐helices (residues 1–10 α_1_, 22–34 α_2_, 49–51 α_3_, 67–76 α_4_, 90–97 α_5_, 98–120 α_6_, 153–174 α_7_ and the C‐terminal residues 227–239 α_9_, 241–244 α_10_ and 293–301 α_12_). The parallel β‐strands are sandwiched on one side by α‐helices α_1_, α_2_, α_3_, α_9_, α_10_ and α_12_ and on the other by α‐helices α_4_, α_5_, α_6_ and α_7_. The characteristic SDR superfamily [24, 31, 32, 34] extended coenzyme‐binding motif ***G**‐X‐X‐**G**‐X‐X‐**G*** is located on the short loop between β_1_ and α_2_ (Fig. 2C; coenzyme loop 1; residues 18–24) with sequence ***G**‐G‐A‐**G**‐C‐V‐**G***. Three additional loops make contact with the bound NADH including residues 43‐48 (coenzyme loop 2) between β_2_ and α_3_, residues 63‐66 (coenzyme loop 3) between β_3_ and α_4_ and residues 86–90 (coenzyme loop 4) between β_4_ and α_5_. An extended loop (residues 130–153) also straddles both the substrate and coenzyme‐binding domains between β_5_ and α_7_. The smaller Mth375 C‐terminal domain is comprised of six short β‐strands (formed by residues 184–186 β_7_, 212–216 β_8_, 220–222 β_9_, 225–226 β_10_, 256–257 β_12_ and 274–278 β_13_) and three large α‐helices (formed by residues 197–207 α_8_, 258–269 α_11_ and 308–329 α_13_). A catalytic motif common amongst SDRs (***Y**‐X‐X‐X‐**K***) [24, 31] is located on α_7_, with sequence ***Y**‐Q‐V‐T‐**K*** (residues 155–159). Interactions with UDP and Mth375 are depicted in Fig. 2D and are maintained in part by secondary structure elements α_7_, α_8_, β_7_, β_8_, β_9_, α_11_ and α_5_, and the active site loop between β_9_ and α_12_.

Mth375 is a dimer (Fig. 2B) with a total buried surface of 6689 Å^2^ and a dissociation complex ΔG^o^_diss_ of 12.4 kcal·mol^−1^ as calculated by PISA [35]. The dimeric structure is maintained via hydrogen bonds between residues present on the adjacent extended loops (EX‐L) between β_5_ and α_7_ which stack along opposing helices (α_7_). This includes the side chain of Asn167 (ND2) and the main chain carbonyl of Ile149 (O; 2.9 Å), and the side chain of Asn171 (ND2) which forms hydrogen bonds to the main chain of Leu151 (O; 3.0 Å) and the side chain of Ser150 (OG; 2.9 Å). We also observe identical secondary structure elements α_6_ and α_7_ from each monomer stacking along each other in an antiparallel fashion where multiple hydrophobic contacts are made forming a singular water‐excluding patch predominantly between Leu102, Leu107, Ile109, Leu110, Leu113, Val157, Leu160, Leu161, Leu164, Tyr165, Tyr168 and Phe169.

Mth375_NU revealed interpretable density for the molecule NAD(H). Its presence was not unexpected and is consistent with the conservation of coenzyme binding within epimerases such as GalE [33] by the NAD(H)‐binding loop (residues 43–48 in Mth375) forming interactions with the adenine and ribose moiety of NAD(H) (Fig. 2C). The competitive exclusion of the NAD(H) coenzyme by AMP in Mth375_AU is understandable as both molecules form equivalent interactions with the enzyme with their equivalent moieties. The conserved coenzyme‐binding motif (sequence ***G**‐G‐A‐**G**‐C‐V‐**G***) makes hydrogen bond contacts with the pyrophosphate bridge of NADH and main chain amines of Cys22 (N) and Val23 (N). The NAD(H)‐binding loop forms numerous hydrogen bond contacts with the NAD(H) ribose sugar including the side chain carboxylate of Asp42 (OD1), the side chain of Ser47 (OG), and with the main chain of Ser45 (N) and Ser46 (N). The side chain of Ser46 also makes an additional contact with the pyrophosphate bridge (OG) as does the side chain of Arg196 (NH1). The adenine moiety forms hydrogen bond contacts with the Ile64 main chain (N) and side chain of Asp64 (OD1) while the remaining bonds are between the ribosylnicotinamide moiety of NAD(H) and residues Ser127 (OG), Leu85 (O), Lys159 (NZ), Tyr155 (OH) and Val185 (N).

A single molecule of UDP was bound to each Mth375 complex (Fig. 2D). The UDP diphosphate moiety forms a number of hydrogen bond interactions with Asn91 (ND2), Asn184 (ND2), Arg222 (NE and NH2), Arg281 (NH1 and NH2) and Val198 (N), and water molecules including O98, O29, O109 and O146. The UDP ribose hydroxyls form hydrogen bond contacts with the side chain and main chain of Thr215 (OG1 and O) and water molecules O33, O135 and O52. The carbonyl groups present on the uracil moiety make hydrogen bond interactions with waters O64 and O141, the side chain hydroxyl and main chain of Thr215 (OG1 and N), while the 2′‐amide forms a hydrogen bond with Pro213 (O).

### Probing the sugar‐binding domain of Mth375

**Fig. 3:**
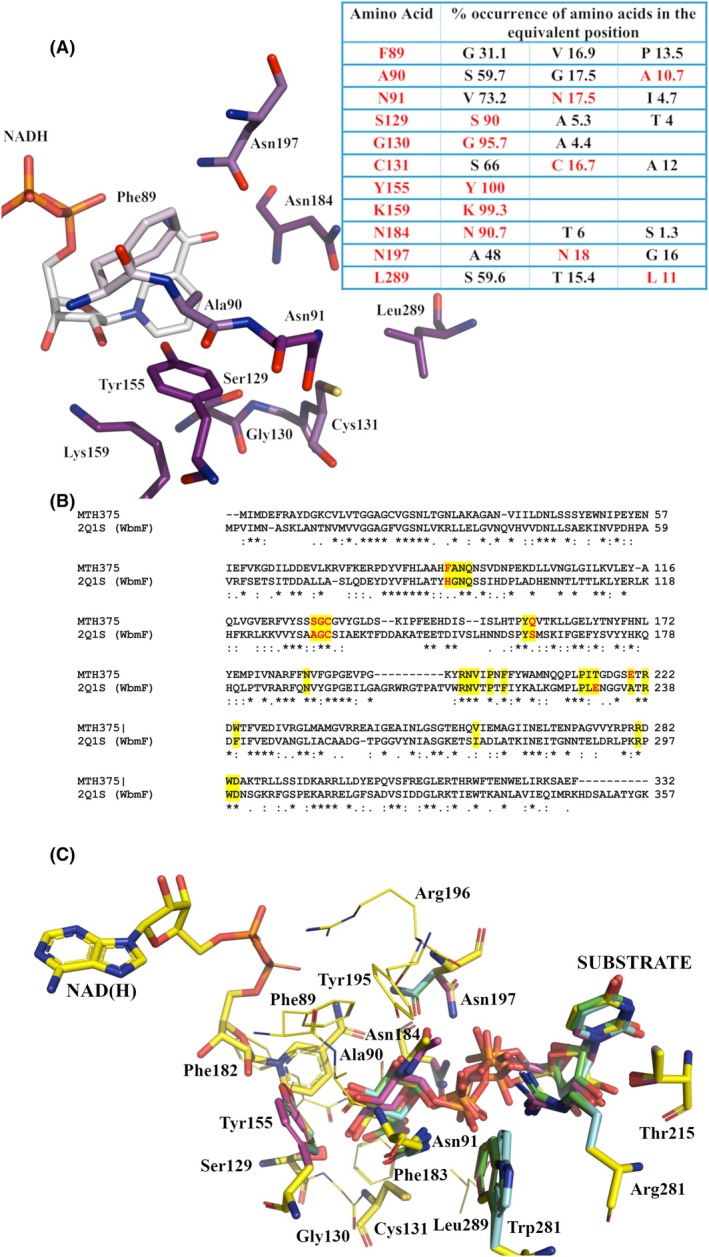
The substrate binding domain of Mth375. (A) ConSurf depiction and analysis of Mth375 sugar‐binding domains [73]. The table details the top amino acids in terms of % conservation (matching residues are coloured red) for each position. (B) Clustal Omega (0.1.2.4) [38] sequence alignment of WbmF enzymes Mth375 (accession number O26475) and PDB: 2Q1S (*Bordetella bronchiseptica*; accession number O87989). Active site residues within 5 Å of the bound substrate are coloured yellow, ‘*’ indicates identical residues, ‘:’ and ‘.’ indicate sequence similarity. (C) Potential substrates docked into the Mth375 crystal structure, including HP7 (carbon atoms coloured yellow), UPG (blue), UGA (green) and UD1 (pink). Compounds were docked using GOLD [39] with the top ranked poses for each compound depicted. Structure figures were prepared using PyMOL version 2.5.8.

Some epimerases possess a larger active site to incorporate and rotate an acetylated saccharide moiety during catalysis, which is not the case for the smaller UDP‐galactose/glucose substrates of GalE. When comparing the signature key residues of WbpP from *P. aeruginosa* (PDB: 1SB8; Gly102, Ser103, Val104, Tyr166, Asn195, Ala 209 and Ser306) [36] with Mth375, significant differences arise. The corresponding residues in Mth375 include Phe89, Ala90, Asn91, Tyr155, Asn184, Asn197 and Leu289. Significant clashes would likely occur between the UDP‐GlcNAc acetyl group during 4‐epimerisation with the sidechains including Leu289, Phe89, Asn91, Asn197 and Cys131. ConSurf analysis of the sugar‐binding domain indicates that these residues are the most variable in the active site (see Fig. 3A) and often the most common amino acid has a smaller sidechain. Overall, 4‐epimerisation of an acetylated sugar is unlikely to occur for Mth375.

When comparing GalE enzymes such as those from *Burkholderia pseudomallei* (PDB: 3ENK) the bound UPG molecule makes several interactions with key active site residues including Lys88, Ala89, Val90, Tyr152, Asn182, Asn202 and Cys302, which is dissimilar to the architecture of Mth375 (Phe89, Ala90, Asn91, Tyr155, Asn184, Asn197 and Leu289). Structural alignment of both enzymes shows that the hydroxyl groups of UPG do form several potential hydrogen bond contacts with multiple Mth375 residues; however, an observed steric clash with Asn91 would require a large rotameric movement of the sidechain to allow the 4‐epimerisation reaction mechanism to proceed.

**Fig. 4:**
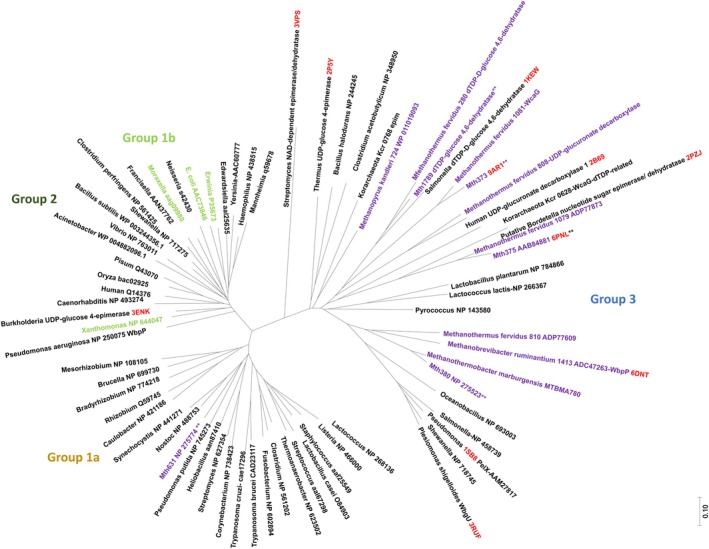
A phylogenetic tree of archaeal SDR's. Neighbour‐Joining [74] phylogenetic tree of methanogen 4‐epimerases together with Group 1‐3 proteins from the phylogenetic analysis by Ishiyama *et al*. [36]. Sequences for crystal structures of relevant 4‐epimerases discussed in this text are also included for reference (highlighted in red text). Group names are coloured to match those of Ishiyama *et al*. [36] using the following colour‐coding: Group 1a (mustard); Group 1b (light green); Group 2 (dark green); and Group 3 (blue). Group 1b sequences are shown in light green text and are interspersed with Group 2 sequences. Methanogen epimerase branches are shown with lavender text, and epimerases that had their structures determined or modelled in this study are indicated with asterisks (**). Seventy‐two amino acid sequences were downloaded from the non‐redundant NCBI or RCSB databases and imported into MEGA7 [75]. The sequences were aligned using Clustal Omega (0.1.2.4) [38] and a Neighbour‐Joining phylogenetic tree [74] constructed in MEGA7 using default parameter settings [75]. The tree is drawn to scale, with branch lengths in the same units as those of the evolutionary distances used to infer the phylogenetic tree. The evolutionary distances were computed using the Poisson correction method [78] and are in the units of the number of amino acid substitutions per site. All positions containing gaps and missing data were eliminated. There was a total of 208 positions in the final dataset.

The pliable active site architecture of this enzyme group explains why Mth375 shares structural homology with multiple epimerase classes shown in Table 2, where a topology‐based assessment of Mth375_NU using CLICK [37] was undertaken. The structural homologues of Mth375 included the bacterial 4‐epimerase subfamilies known as WbgU (PDB: 3RUF; EC5.1.3.7), GalE (PDB: 2P5Y and PDB: 3KO8; EC5.1.3.2), human UDP‐glucuronate decarboxylase (PDB: 2B69; EC4.1.1.35), bacterial WbpP (PDB: 1SB8; [36]), bacterial NAD‐dependent epimerase/dehydratase (PDB: 3VPS; EC 4.2.1) and a likely 3,5‐epimerase WbmF belonging to the pathogenic bacteria *Bordetella bronchiseptica* (PDB: 2Q1S and PDB: 2PZJ; [34]). Sequences of selected structures from Table 2, with varying activities, were submitted to the Clustal Omega (0.1.2.4) alignment server [38] and residues within 5 Å of the Mth375 UDP molecule were highlighted. The highest active site and overall sequence identity is with *Bordetella bronchiseptica* WbmF (PDB: 2Q1S; Fig. 3B, Table 2) which clades in Group 3 (PDB: 2PZJ; Fig. 4). Enzymes in this class present a strong preference for an acetylated substrate, for example, *B. bronchiseptica* WbmF catalyses the 3,5‐epimerisation required in the conversion of UDP‐2,3‐diacetamido‐2,3‐dideoxy‐d‐mannuronic acid to UDP‐2,3‐diacetamido‐2,3‐dideoxy‐l‐galacturonic acid [34]. Furthermore, a sequence and structural comparison of the potential sugar‐binding residues of Mth375 with UDP bound WbmF (PDB: 2Q1S) and GalE bound to UPG (PDB: 3ENK) reveals the following active site Mth375/WbmF/GalE fingerprints: Phe89/His90/Lys88, Ala90/Gly91/Ala89, Asn91/Asn92/Val90, Ser129/Ala131/Ser128, Cys131/Cys133/Thr130, Tyr155/Tyr161/Tyr152, Asn184/Asn190/Asn182, Asn197/Asn213/Asn202, Arg281/Arg296/Arg295 and Trp283/Trp298/Gly297. WbmF enzymes have a conserved catalytic cysteine (Cys133) in an identical position as the modified cysteine of Mth375 (Cys131); however, it is structurally disordered in the WbmF crystal structure as are residues Ser134‐Thr146. This disorder may be the reason for the large deviation in secondary structure elements around the active site of WbmF and the reason we observe WbmF Arg296 and Trp298 Cα carbons 18 Å away from Mth375 Arg281 and Trp283 Cα atoms. WbmF active sites also contain a second positionally conserved basic side chain [34], equivalent to Mth375 Asn197 and not in the position occupied by the hydrophobic residue Phe89. Mth375 unlike other previously described WbmFs possesses the catalytic Ser129 suggesting that it could act as an oxidoreductase and/or a 3,5‐epimerase [34].

**Table 2:** A topology‐based assessment of Mth375 structural homologues utilising CLICK [37].

Rank	Homologous structure	Z‐score	Structural overlap (%)	RMSD (Å)	#identical residues	Homologue description	Gene name/mutation^a^	EC^a^	Substrate^a^	Organism
1	3RUF‐A	9.02	93.71	1.28	92	WbgU	WbgU	5.1.3.7	UDP	*Plesiomonas shigelloides*
2	2P5Y‐A	8.75	92.93	1.28	90	UDP‐glucose 4‐epimerase	TTHA0591	5.1.3.2	–	*Thermus thermophilus*
3	2B69‐A	8.31	92.72	1.39	92	UDP‐glucuronate decarboxylase 1	UXS1	4.1.1.35	UDP	*Homo sapiens*
4	1SB8‐A	8.24	91.92	1.42	88	WbpP	–	5.1.3.7	UD2	*Pseudomonas aeruginosa*
5	3VPS‐A	7.93	93.40	1.51	79	NAD‐dependent epimerase/dehydratase	TunA	4.2.1	UD1	*Streptomyces chartreuses*
6	3KO8‐A	7.92	92.59	1.52	71	UDP‐galactose 4‐epimerase	GalE	5.1.3.2	–	*Pyrobaculum calidifontis*
7	2Q1S‐A	7.62	85.03	1.35	109	Putative nucleotide sugar epimerase/dehydratase	WbmF	–	–	*Bordetella bronchiseptica*
8	3ENK‐A	7.43	87.43	1.44	80	UDP‐glucose 4‐epimerase	GalE	5.1.3.2	UPG	*Burkholderia pseudomallei*
9	1EK6‐A	7.34	89.22	1.54	80	UDP‐galactose 4‐epimerase	GalE	5.1.3.2, 5.1.3.7	UPG	*Homo sapiens*
10	1KEW‐A	7.24	91.02	1.53	84	dTDP‐glucose 4,6‐dehydratase	RmlB	4.2.1.46	TYD	*Salmonella typhimurium*
11	4LIS‐A	7.21	87.13	1.49	90	UDP‐galactose 4‐epimerase	GalE	5.1.3.2	UPG	*Aspergillus nidulans*
12	1OC2‐A	7.21	90.12	1.56	84	dTDP‐glucose 4,6‐dehydratase	RmlB (RfbB)	4.2.1.46	TDX	*Streptococcus suis*
13	1R6D‐A	7.20	90.99	1.58	91	dTDP‐glucose 4,6‐dehydratase	desIV/D128N, E129Q	4.2.1.46	DAU	*Streptomyces venezuelae*
14	2PK3‐A	7.18	89.64	1.59	66	GDP‐6‐deoxy‐d‐lyxo‐4‐hexulose reductase	RMD	1.1.1.281	GDD	*Aneurinibacillus thermoaerophilus*
15	1UDC‐A	7.00	86.83	1.54	82	UDP‐galactose 4‐epimerase	GalE	5.1.3.2	UFM	*Escherichia coli*

^a^ As annotated in the RCSB (https://www.rcsb.org/).

To investigate potential substrate binding of Mth375, docking analysis was performed for the following molecules UDP‐*N*‐acetylglucosamine (UD1), UDP‐glucose (UPG), UDP‐glucuronic acid (UGA) and UDP‐*N*‐acetyl‐d‐glucosaminuronate (HP7) on NADH and UDP bound Mth375 using the *in silico* docking software GOLD [39]. The Asn91, Asn184 and Asn197 side chains were allowed the most rotameric freedom during docking to accommodate binding of the larger sugar bound UDP molecules, with the resulting top ranked poses depicted in Fig. 3C. The binding poses of the pyranose sugars of each potential substrate were near identical, and the large rotameric movements of Asn184 and Asn197 allowed for the best induced fit and reduced clash scoring for each molecule. UD1 scored best followed closely by UPG, ranked first and second amongst the docked poses, while HP7 and UGA ranked tenth and nineteenth, respectively. The orientation of UPG closely mimicked that seen in 1EK6, which could suggest that 4‐epimerisation is possible but would require a large rotameric movement of Asn91. As previously stated, however, this would not be possible for the even larger acetylated or carboxylated substrates. The lower rankings of HP7 and UGA poses may be due to interactions of the carboxylate atop a small hydrophobic pocket created in part by residues Cys131, Phe183 and Leu289; however, hydrogen bond interactions with the sidechain hydroxyl of Ser129 and the sidechain carbonyl of Asn184 may ameliorate any nonoptimal binding. Similarly, the hydroxyl of UD1 in the same position can make hydrogen bond interactions with the same residues. Overall, each molecule docked into Mth375 produced reasonable binding of near identical poses, making a definitive annotation of activity inconclusive; however, we can eliminate UD1 epimerisation.

## Crystal structures of Mth373

**Fig. 5:**
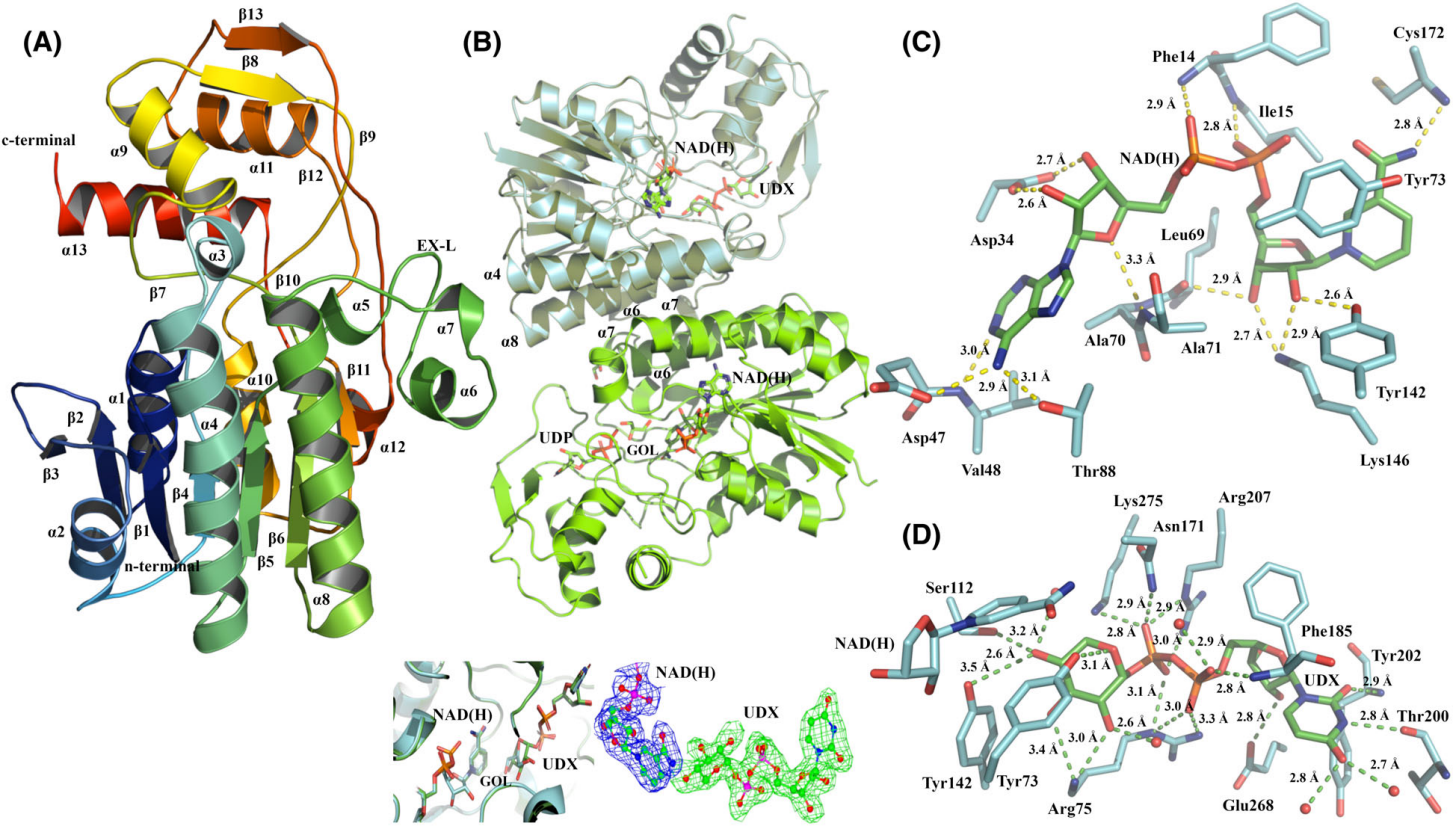
Structures of *M. thermautotrophicus* ΔH Mth373. (A) A ribbon representation of methanogen Mth373_NX (EC 4.1.1.35/4.2.1.46) with secondary structural elements labelled. The structure is colour‐ramped from the N terminus (blue) to C terminus (red). (B) Dimeric ribbon representation of Mth373_NX. Important secondary structure elements α4, α8, α6 and α7 are labelled. Inset bottom left: the Mth373_NX monomers are superimposed illustrating the binding modes of GOL and UDX in the active site of the enzyme alongside an omit electron density map (Fo‐Fc) of bound UDX (in green). (C) The coenzyme (NAD(H)) binding domain of Mth373_NX (carbon atoms in cyan). (D) The active site of Mth373_NX with bound UDX (carbon atoms green). Relevant residues and water molecules within 4 Å of UDX and NAD(H) are depicted. Polar contacts with distances are indicated as dashed lines. All figures were prepared using PyMOL version 2.5.8.

Mth373 expressed with a yield of 4.1 mg·L^−1^ culture and eluted as a single peak from nickel‐affinity purification with purity appropriate for protein crystallisation (in excess of 95% as assessed by SDS/PAGE). Mth373_NU crystallised in the orthorhombic space group *P 2 2 21* with unit cell parameters *a* = 54.5 Å, *b* = 81.4 Å, *c* = 152.4 Å, α = β = γ = 90.0° and solvent content 46% (Matthews coefficient of 2.29 Å^3^·DaÅ^3^·Da^−1^). Mth373_NX crystallised in space group *H 3 2* with unit cell parameters *a* = 139.3 Å, *b* = 139.3 Å, *c* = 226.9 Å, α = β = 90° and γ = 120° and a solvent content of 59% (Matthews coefficient of 3.03 Å^3^·Da^−1^; see Table 1 for diffraction data and refinement, Mth373_NU and Mth373_NX statistics). Both Mth373 complexes crystallised as a dimer within the asymmetric unit (Fig. 5A,B), with an average total buried surface of 8631.7 Å^2^ and a calculated dissociation complex ΔG^o^_diss_ of 20.7 kcal·mol^−1^ [35]. Electron density maps for both Mth373 structures displayed unbroken electron density throughout the main chain of each 311‐residue monomer apart from Glu60 and a few instances of side chain disorder, which for Mth373_NX was predominantly at the C terminus. Both complexes have interpretable electron density for a molecule of NADH in the N‐terminal coenzyme‐binding domain (Fig. 5C) of each monomer and a molecule of UDP in each of the active site domains of Mth373_NU, but in only one of the active site domains of Mth373_NX. Co‐crystallisation with UGA failed to show binding to the structure; however, the active site domain of Mth373_NX contained electron density indicative of a bound uridine‐5′‐diphosphate‐xylopyranose (UDP‐xylose, UDX; Fig. 5D). For both Mth373 complexes, the UDP and NADH molecules superimpose in an identical manner, so results and discussion will centre exclusively on Mth373_NX. The electron density map is depicted in Fig. 5 for the bound UDX molecule.

Mth373, like the aforementioned Mth375 and other archaeal, bacterial and eukaryotic 4‐epimerases [24, 26, 33] of the SDR superfamily, shares a common architecture with an N‐terminal nucleotide‐binding domain predominantly formed by a modified Rossmann‐fold motif (residues 1–168), binding NADH, and a C‐terminal active site domain that binds the UDP‐sugar substrate (residues 169–311; Fig. 5A). The N‐terminal domain is formed by seven parallel β‐strands (residues 4–10 β_1_, 29–34 β_2_, 42–46 β_3_, 64–69 β_4_, 105–110 β_5_, 163–168 β_6_ and by C‐terminal domain residues 230–234 β_11_) and ten α‐helices (residues 13–26 α_1_, 50–61 α_2_, 75–80 α_3_, 82–104 α_4_, 112–117 α_5_, 126–130 α_6_, 133–137 α_7_, 140–161 α_8_ and C‐terminal domain residues 213–225 α_10_, and 280–285 α_12_). The parallel β‐strands are sandwiched on one side by α‐helices α_2_, α_4_, α_5_, α_6_, α_7_ and α_8_ on the other by α‐helices α_1_, α_10_ and α_12_. The extended coenzyme‐binding motif ***G**‐X‐X‐**G**‐X‐X‐**G*** is located on the short loop between β_1_ and α_1_ (coenzyme loop 1; residues 10–16) with sequence ***G**‐G‐A‐**G**‐F‐I‐**G***. Coenzyme loop 2 in Mth373 is shorter than in Mth375, making fewer contacts with the coenzyme (residues 34–36) and is located between β2 and β3. Coenzyme loop 3 is formed between β_3_ and on α_2_ (with residues 46–49) and coenzyme loop 4 falls between β_4_ and on α_3_ (residues 69–74). An extended loop (residues 112–124) that incorporates a short alpha helix (α_5_) also straddles both the substrate and coenzyme‐binding domains and is formed between β_5_ and α_6_. The C‐terminal domain of Mth373 is comprised of six very short β‐strands (formed by residues 171–173 β_7_, 199–203 β_8_, 205–207 β_9_, 210–211 β_10_, 242–243 β_12_ and 261–265 β_13_) and three large α‐helices (formed by residues 184–195 α_9_, 244–256 α_11_ and 293–309 α_13_). The catalytic motif of the enzyme is located on α_8_, with sequence ***Y**‐A‐I‐T‐**K*** (residues 142–146, α_8_) [24, 31]. Interactions with the UDP‐bound sugar are depicted in Fig. 5D and are maintained in part by secondary structure elements α_5_, α_8_, α_9_, α_11_ and β_8_, and the active site loop between β_4_ and α_3_. Overall, the dimeric structure of Mth373 is formed via several hydrogen bond networks including the interactions between residues of the adjacent helices of α_6_ and α_7_ and via the antiparallel stacking of helices α_8_ and α_4_ (Fig. 5B). This latter interaction also forms a water‐excluding hydrophobic patch created by residues Ile144, Ala148, Leu151 and Met152 on α_8_ and Val90, Ile91 and Trp86 on α_4_. Additional salt bridges between monomers are maintained primarily by the side chain of Glu79 (OE1, α_3_) and the side chain of Lys94 (NZ; 2.80 Å, α_4_) and Arg98 (NH2; 3.26 Å, α_4_), and the side chains of Asp141 (OD1, α_8_) and Lys94 (NZ; 2.81 Å, α_4_).

The Mth373 to NAD(H) coenzyme molecular interactions are depicted in Fig. 5C. The conserved coenzyme‐binding motif (sequence ***G‐G‐A‐G‐F‐I‐G***) main chain amines Gly13 (N), Phe14 (N) and Lys183 (N) make hydrogen bond contacts with the NADH pyrophosphate bridge and ribose sugar. The shortened NAD(H)‐binding loop interacts with the NADH adenine via a π‐stacking interaction (Leu35), while the NADH ribose hydroxyls form hydrogen bond contacts with Asp34 (OD1 and OD2). The ribose ring oxygen of the adenosine moiety forms an additional hydrogen bond interaction with the main chain of Ala71 (N) while the adenine base forms hydrogen bond contacts with the main chain of Val48 (N), the side chain of Asp47 (OD1) and the side chain of Thr88 (OG1). The ribosylnicotinamide forms hydrogen bond contacts via Leu69 (OÅ), Tyr142 (OH), Lys146 (NZ) and Cys172 (N). A π‐stacking interaction is also observed via the mostly conserved side chain of Tyr73, which is immediately adjacent to the catalytic Tyr142 and the nicotinamide ring of NADH.

A single molecule of UDP was bound to each active site of the Mth373_NU dimer. For Mth373_NX, one monomer contained UDP and additional density consistent with a single molecule of glycerol in the sugar‐binding domain, while the second monomer contained electron density refined as uridine‐5′‐diphosphate‐xylopyranose (UDP‐xylose, UDX; Fig. 5). Mth373 apo preparations and subsequent crystallisation always resulted in complexes with clearly bound coenzyme and little to no clear electron density corresponding to the presence of bound substrate (results not shown). It is unknown if the observed UDP‐xylose (UDX) molecule bound in Mth373_NX is a result of enzymatic activity or decarboxylation of bound UDP‐glucuronic acid (UGA) during synchrotron data collection, or a combination of the two in a mechanism where the Mth373 active site has strained the UDP‐glucuronic acid carboxylate so that it is more prone to radiation effects [40]. Similarly, we cannot explain why one active site domain of Mth373_NX possesses a single UDP molecule and glycerol (see inset panel of Fig. 5B). Glycerol is not present in the nickel‐affinity purification steps; it is not in the storage buffer, mother liquor or cryoprotectant and was not seen in other crystallisation experiments with the enzyme.

The diphosphate moiety of UDX forms several hydrogen bond interactions including Asn171 (ND2), Lys275 (NZ), Arg207 (NE and NH2), Arg75 (NH2and NE) and Phe185 (N) and water molecule O27. The uridine ribose hydroxyls form hydrogen bond contact with the side chain carboxylate of Glu268 (OG), while the carbonyl groups present on the uracil moiety make hydrogen bond interactions with Tyr202 (N) and waters O587 and O649, while the 2′‐amide forms a hydrogen bond with Thr200 (O). The hydroxyls of the UDX UDP‐xylose make hydrogen bond contacts with Arg75 (N), the side chain hydroxyls of Ser112 (OG), Tyr142 (OH) and water molecules O528 and O555, while the oxygen of the pyran ring forms a hydrogen bond contact with the side chain hydroxyl of Tyr73 (OH).

### Probing the sugar‐binding domain of Mth373

**Fig. 6:**
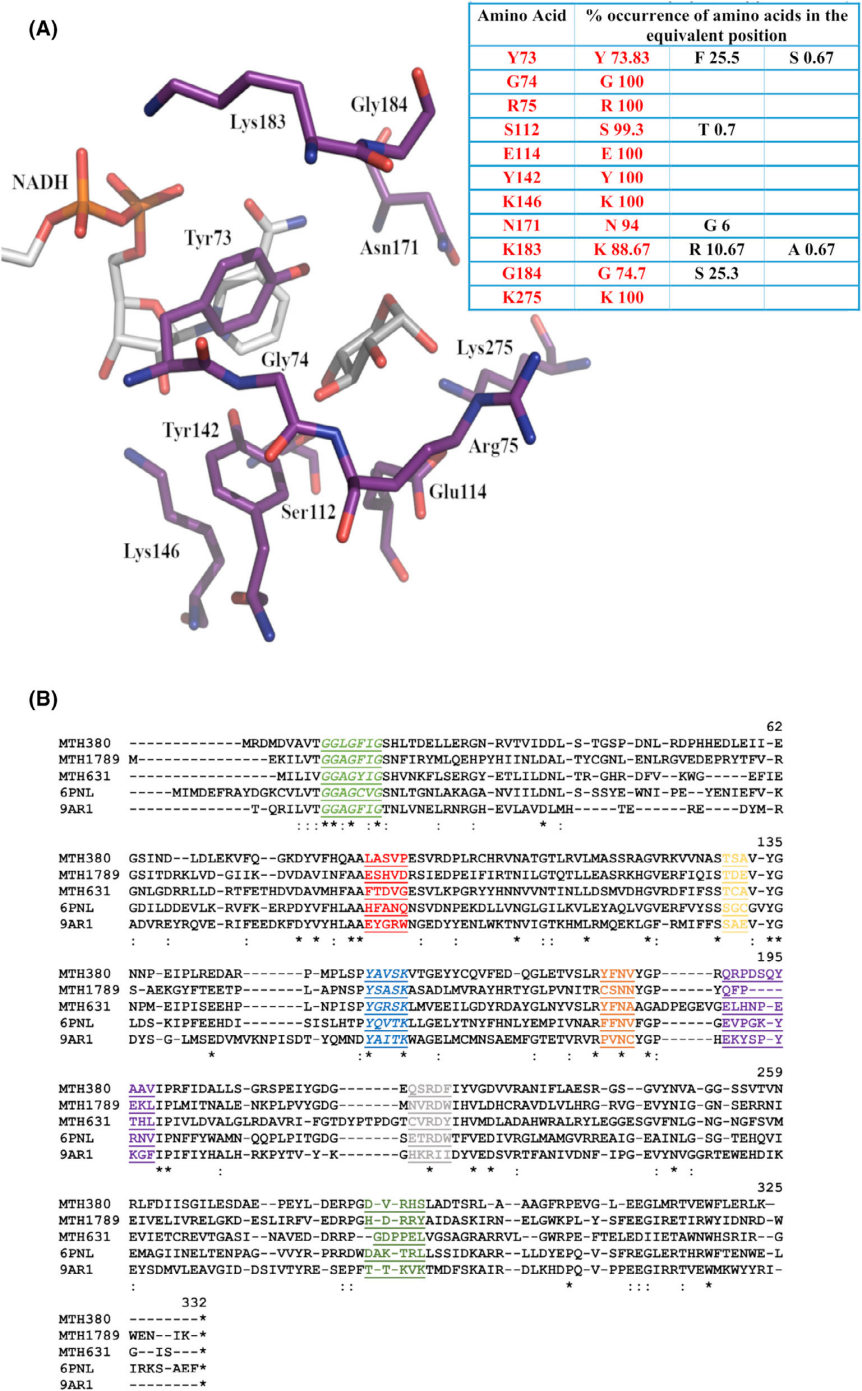
The substrate binding domain of Mth373. (A) ConSurf analysis of Mth373 [73]. The table details the residues in % conservation terms for Mth373 amino acid position within the sugar‐binding domain of the enzyme. The matching conserved residue is shown in red with decreasing conservation values from left to right in columns 2–4. (B) The *M. thermautotrophicus* ΔH enzyme structures Mth380 (accession number O26480), Mth1789 (accession number O27817), Mth631 (accession number O26728), Mth375 (PDB: 6PNL; accession number O26475) and Mth373 (PDB: 9AR1; accession number O26473) were superimposed using SALIGN [72]. The resultant sequence alignment was calculated by Clustal Omega (0.1.2.4) multiple sequence alignment online server [38]. Identical residues are indicated with a ‘*’. The catalytic residues are in blue indicating the characteristic signature sequence *Y‐X‐X‐X‐K* of short‐chain dehydrogenases/reductases, while the coenzyme signature sequence *G‐G‐X‐G‐X‐X‐G* is in green. Each substrate binding domain within 5 Å of a bound UDP‐sugar is also highlighted and shaded to match groups previously delineated and colour coded (red, mustard yellow, blue, orange, purple, grey, dark green) [36, 52]. Numbering is based on the PDB: 6PNL crystal structure. Structure figures were prepared using PyMOL version 2.5.8.

Mth373 clades with the *Salmonella enterica* structure PDB: 1KEW annotated as a dTGD (EC 4.2.1.46; [41]), the human UDP‐glucuronate decarboxylase PDB: 2B69 (EC4.1.1.35) and the WcaG family of epimerases. The active site of Mth373 depicts the largely conserved catalytic triad [42] of Lys146, Tyr142 and Ser112 with ConSurf analysis of residues immediately around the xylopyranose sugar of UDX also showing a remarkable level of conservation (see Fig. 6A). The only real exception being a residue immediately adjacent to the catalytic triad, Tyr73 (typically conserved as phenylalanine) and Gly184 (typically conserved as serine). These residues are present on flexible loops known to contribute to substrate binding [43] including the immediate loop region preceding α3 (residues 73–76) and an elongated loop between β7 and α9 (residues 174–183), which may indicate a paired substitution of function for the two residues to stabilise substrate binding with the hydroxyl group now provided by Tyr73 instead of serine as found in homologues. The presence of this second tyrosine immediately adjacent to the catalytic domain and the nicotinamide of the NADH is a unique feature of the Mth NS‐SDRs, as is the positioning of its sidechain hydroxyl within 3.0 Å and 3.3 Å of the C4 and C5 positions of the UDX xylopyranose moiety. However, other SDRs involved in UGA decarboxylation reactions, notably UDP‐d‐apiose/UDP‐d‐xylose synthase from *Arabidopsis thaliana* (UAXS; [44], PDB: 6H0N), human UDP‐α‐d‐xylose synthase (UXS; [43]; PDB: 4GLL and PDB: 2B69) and ArnA, a Gram‐negative bacteria UGA decarboxylase involved in lipopolysaccharide synthesis ([45]; PDB: 2BLL) do possess several key similarities (summarised in Table 3) outside of the catalytic residues such as a second active site tyrosine. The equivalent tyrosines of PDB: 4GLL, PDB: 2BLL and PDB: 6H0N are found within a helix equivalent to the α3 helix of Mth373, while Tyr73 of Mth373 is present on the preceding loop. This is the same location as Cys100 of PDB: 6H0N, a key residue involved in substrate proton transfer and glucuronic acid ring opening during catalysis [44]. This process occurs prior to decarboxylation which is followed by either sugar ring contraction to form a UDP‐apiose or without contraction to form UDP‐xylose. Other identically positioned residues between these homologues include asparagine and glutamine residues, with the latter playing an important role in the relay of protons during catalysis driving the decarboxylation reaction [44, 45]. PDB: 4GLL, PDB: 2BLL and PDB: 6H0N have two identically positioned arginines with one of these replaced by Lys275 (in contact with the UDX diphosphate moiety) in Mth373. Arg75 present on Mth373 α3 replaces the interactions observed in the second conserved Arg site, making a larger number of hydrogen bond interactions with the substrate than that seen in PDB: 4GLL, PDB: 2BLL and PDB: 6H0N. Arg75 also makes a salt bridge with Glu268, configuring/stabilising the Mth373 active site. Overall, the most likely functional assignment for Mth373 is as a UDP‐xylose synthase (EC4.1.1.35) based on sequence analyses and the presence of UDP‐xylose in the Mth373_NX structure, with Tyr73 facilitating proton transfer and/or stabilising the transition state of the substrate, combined with Glu114, to drive UDP‐xylose formation, and that the oxidative decarboxylation of the UGA does not likely involve cleavage of the glucuronic acid ring as is seen for PDB: 6H0N.

**Table 3:** Comparison of UDP‐xylose synthases, catalytic and selective active site residues along with the proposed catalytic mechanisms.

Mth373	2B69/4GLL	2BLL	6H0N
*M. thermautotrophicus ΔH*	*Homo sapiens*	*Escherichia coli*	*Arabidopsis thaliana*
UDP‐xylose synthase	UXS	ArnA	UAXS
EC4.1.1.35	EC4.1.1.35	EC4.1.1.35	
Reaction	Reaction	Reaction	Reaction
UGA oxidative decarboxylation	UGA oxidative decarboxylation	C4’ hydride abstraction (oxidation) and subsequent decarboxylation	NAD(H) linked oxidation and reduction Aldol cleavage/Sugar ring opening UGA decarboxylation aldol‐linked substrate contraction and furanosyl production/aldol‐linked pyranosyl production
Catalytic triad	Catalytic triad	Catalytic triad	Catalytic triad
Lys146	Lys151/235	Lys467	Lys189
Tyr142	Tyr147/231	Tyr463	Tyr185
Ser112	vThr118/202	Thr432	Thr139
Aligned signature residues	Aligned signature residues	Aligned signature residues	Aligned signature residues
–	Tyr84/168	Tyr398	Tyr105
Glu114	Glu120/204	Glu434	Glu141
Arg75^a^	Arg144/228	Arg460	Arg182
Lys275	Arg277/361	Arg619	Arg341
Asn171	Asn176	Asn492	Asn214
Tyr73	Ala79/Ala163	Ala393	Cys100
Ala113	Ser119/Ser203	Ser433	Cys140

^a^ Not in identical sequence position.

## Probing the sugar‐binding domain of Mth1789, Mth631 and Mth380

Pseudomurein cell wall production requires a number of enzyme activities to convert sugar precursors to the glycan component of pseudomurein [15, 16, 18] with *M. thermautotrophicus* ΔH possessing several enzymes that could play these roles. These include Mth373 and Mth380 which are in two juxtaposed gene clusters (Mth370‐Mth378 and Mth379‐Mth381) annotated as encoding proteins involved in extracellular carbohydrate metabolism. The presence of Mth373 and Mth380 in these two *M. thermautotrophicus* gene clusters, along with Mth1789 and Mth631, strongly suggests that they play differing or overlapping roles in extracellular carbohydrate metabolism. Substrate specificity within the NS‐SDRs is primarily the result of the dynamic protein flexibility seen in residues coordinating their sugar substrates and catalytic intermediates, with as little as one amino acid change potentially altering the substrate specificity and classification of the enzyme. Structural alignment of the Mth1789, Mth631 and Mth380 models, combined with the Mth375 and Mth373 crystal structures demonstrates this, with the Tyr and Lys catalytic residues (Fig. 6B italicised blue region) being the only strictly conserved active site residues across all these *M. thermautotrophicus* enzymes, apart from an Asn (orange region). A catalytic Ser is observed in Mth373 and Mth375 and is a Thr in Mth1789, Mth631 and Mth380 (mustard yellow region).

A number of studies [36, 46, 47, 48] have identified common catalytic residues amongst UDP‐glucose 4‐epimerases (the S/T, Y, K triad; residues Ser129, Tyr155 and Lys159 in Mth375) and established the idea of ‘gatekeeper’ residues that mediate the propensity for acetylated or nonacetylated UDP‐sugar substrates by significantly changing the volume of the sugar‐binding domain. This includes studies by Guo *et al*. [48] which showed that mutagenesis of the *E. coli* UDP‐glucose 4‐epimerase Ser306 to a tyrosine reduced activity with nonacetylated substrates by fivefold and completely removed activity with acetylated substrates. Furthermore, 4‐epimerases can be delineated into groups with differential preferences for their substrates [33, 36, 49]. Group 1 enzymes prefer nonacetylated substrates, Group 2 enzymes do not show a strong preference for either acetylated or nonacetylated substrates, while Group 3 shows a preference for acetylated substrates [36]. A phylogenetic tree of hexose 4‐epimerase sequences was constructed from Groups 1–3 [36] together with methanogen sequences and those for some recently described crystal structures (Fig. 4) in order to inform the substrate specificity of the Mth enzymes. Our updated analysis shows the epimerase sequences separating into three main clades similar to those described by Ishiyama [36]. Mth631 is embedded in Group 1a sequences which supports its designation as a GalE epimerase. Mth1789 clades closely with PDB: 1KEW in Group 3 indicating it to be a dTDP‐d‐glucose 4,6‐dehydratase. Mth380, also in Group 3, clades closely with the previously characterised *Methanobrevibacter ruminantium* 1413 (PDB: 6DNT) [26] which is designated a UDP‐*N*‐acetylglucosamine 4‐epimerase (WbpP; EC5.1.3.7).

Summarised in Table 4 are the sugar‐binding and active site motifs of the *M. thermautotrophicus* enzymes that were modelled using Molsoft [50, 51], their functional annotations and model template details, in comparison to the crystal structures of Mth375 and Mth373. Amongst the modelled proteins, only Mth1789 had an identical amino acid fingerprint to other published dTGDs (EC4.2.1.46) [52]. It was modelled using the *Streptomyces venezuelae* dTGD structure (PDB: 1R66; [53]) sharing a 53% structural identity and a 90% active site homology. Mth380 was modelled utilising the crystal structure of *Methanobrevibacter ruminantium* M1 UDP‐*N*‐acetylglucosamine 4‐epimerase (Mru1413; WbpP; EC5.1.3.7; PDB: 6DNT; [26]) with which Mth380 shares the highest sequence identity (55%) of published structures. The signature key residues of Mth380 Ala81, Ser82, Val83, Tyr145, Asn173, Ala187 and Ser276 share high sequence identity with another UDP‐GlcNAc 4‐epimerase, (WbpP; EC5.1.3.7; PDB: 1SB8; [36]) and are identical to PDB: 6DNT [26]. The phylogenetic analysis (Fig. 4) showed that Mth373 clades with a number of UDP‐glucuronate decarboxylases (EC4.1.1.35), dTGD (EC 4.2.1.46; O26473_METTH) and other 3,5‐epimerising oxidoreductases identified as WcaGs that nominally catalyse the formation of GDP‐l‐fucose from GDP‐4‐dehydro‐α‐d‐rhamnose in *E. coli* (GDP‐4‐keto‐6‐deoxymannose‐3,5‐epimerase‐4‐reductase; EC1.1.1.271) [54, 55, 56]. Mth631 is the classical *M. thermautotrophicus* GalE as it does not clade with any enzymes involved in acetylated UDP‐hexose metabolism, and structural and sequence analysis showed that this enzyme best aligns almost exclusively with other GalEs; for example, from *B. anthracis*, (PDB: 2C20) with a 50% total structural identity and an 88% identity amongst the residues that form contacts with the glucose sugar of UPG.

**Table 4:** Sugar‐binding motifs of the NS‐SDRs of *M. thermautotrophicus*. Groups have been previously delineated [36, 52] and colour coded to match.

Substrate binding motifs									
Enzyme	Structure/coenzyme/substrate			Catalytic residues (YX_3_K)				Gate keeper residues	Details
Mth375 [34] Group 3	PDB: 6PMH, 6PNL/NAD/UDP	**_88_ HFANQ_92_**	**_128_ SGC_131_**	**_155_ YQVTK_159_**	**_182_ FFNV_185_**	**_190_ EVPGKYRNV_198_**	**_220_ ETRDW_224_**	**_284_ DAKTRL_289_**	Name; WbmF putative nucleotide sugar epimerase/dehydratase EC–; Substrate: –
Mth373 Group 3	PDB: 8W3U, 9AR1/NAD/UDP, UDX	**_72_ EYGRW_76_**	**_111_ SAE_114_**	**_142_ YAITK_146_**	**_169_ PVNC_172_**	**_177_ EKYSPYKGF_185_**	**_205_ HKRII_209_**	**_271_ TTKVK_275_**	Name; UDP‐xylose synthase EC 4.1.1.35; Substrate: UDP‐glucuronic acid
Mth1789 [52] Group 3	Molecular model (PDB: 1R66)/NAD/TYD, DAU	**_82_ ESHVD_86_**	**_123_ TDE_125_**	**_147_ YSASK_151_**	**_174_ CSNN_177_**	**_182_ QFPEKL_187_**	**_209_ NVRDW_213_**	**_273_ HDRRY_277_**	Name; dTDP– D‐glucose 4,6‐dehydratase EC 4.2.1.46; Substrate: –
Mth380 [36, 76] Group 3	Molecular model (PDB: 6DNT)/NAD/EPZ	**_81_ ASVP_84_**	**_120_ TSA_123_**	**_143_ SPYAVSK_149_**	**_171_ YFNV_174_**	**_179_ QRPDSQYAA_187_**	**_210_ QSRDF_214_**	**_272_ DVRHS_276_**	Name; WbpP EC 5.1.3.7; Substrate: UDP‐*N*‐acetylgalactosamine
Mth631 Group 1a	Molecular model (PDB: 2C20)/NAD/–	**_77_ FTDVG_81_**	**_117_ TCA_120_**	**_142_ YGRSK_146_**	**_169_ YFNA_172_**	**_183_ ELHNPETHL_191_**	**_220_ CVRDY_224_**	**_285_ GDPPEL_290_**	Name; GalE EC 5.1.3.2; Substrate: UDP‐glucose

## Conclusions

We present the first crystal structures of Mth375 a WbmF, and Mth373 (EC4.1.1.35) alongside the structural analyses of Mth380 a WbpP (EC5.1.3.7), Mth1789 a dTGD (EC4.2.1.46) and Mth631 a GalE (EC5.1.3.2) that enable the structural and functional annotation of the *M. thermautotrophicus* ΔH NS‐SDRs. It should be noted that archaea such as *Methanopyrus kandleri* (strain AV19) only possess a single identifiable 4‐epimerase (MK724), which clades with Group 3 enzymes involved in acetylated UDP‐hexose metabolism. It is therefore likely that Group 3 enzymes are involved in *N*‐acetyltalosaminuronic acid production, the unique archaeal sugar moiety of pseudomurein [15, 16, 17, 18, 19]. This is unlikely to include Mth373 decarboxylation reactions. The results presented here suggest that these enzymes have multiple activities to facilitate methanobacterial pseudomurein and capsular polysaccharide production. Future activity studies on these enzymes would confirm the exact/specific role each plays.

## Materials and methods

### Materials

Analytical reagents and chemicals were purchased from Sigma‐Aldrich (St. Louis, MO, USA), Fluka (Ronkonkoma, NY, USA) and Merck (St. Louis, MO, USA). Crystallisation buffers and kits were purchased from Hampton Research (Aliso Viejo, CA, USA) and Molecular Dimensions (Rotherham, UK). *N*‐acetyl‐d‐glucose, *N*‐acetyl‐d‐galactose, *N*‐acetyl‐d‐glucosamine and *N*‐acetyl‐d‐galactosamine were obtained from Sigma‐Aldrich.

### Cloning, expression and purification of Mth375 and Mth373

*Methanothermobacter thermautotrophicus* ΔH (DSM 1053) was obtained from the Leibniz Institute DSMZ (Braunschweig‐Süd, Germany) and grown in BY^+^ media at 65 °C according to Wedlock *et al*. [57]. DNA was isolated using InstaGene Matrix according to the manufacturer's instructions (Bio‐Rad, Hercules, CA, USA). The *Mth375* gene was amplified using forward primer 5′‐CACCTTGATTATGGATGAATTCAGGGCTA and reverse primer 5′‐TTAGAACTCTGCGCTCTTCCTTATG in pET100D (Invitrogen, Carlsbad, CA, USA). The forward primer contained a 5′‐CACC sequence for topoisomerase‐mediated cloning. The positive clone plasmid DNA was sequenced at Massey University Genome Service to confirm the cloned *Mth375* gene sequence was correct. Mth375 was expressed as a recombinant hexahistidine‐tagged protein in *E. coli* BL21‐Rosetta 2 (Novagen, Madison, WI, USA) at 28 °C and purified using protocols previously described [26], with the following changes made. The lysis buffer was 50 mm Tris pH 7.5 containing 2 mm dithiothreitol (DTT), 300 mm NaCl, 10 mm imidazole, 1% (v/v) Triton X‐100, 10 mm MgCl_2_ and 10 μm phenylmethylsulfonyl fluoride (PMSF). The nickel‐affinity equilibration buffer was 50 mm Tris pH 7.5 containing 0.3 m NaCl, 20 mm imidazole and 10% (v/v) glycerol. After purification, the buffer was exchanged by dialysis to 20 mm MOPS pH 7.0 containing 25 mm KCl, 7 mm β‐mercaptoethanol and 5% (v/v) glycerol. The sample was concentrated using a 10 K Vivaspin 20 and 1 mm DTT was added.

The gene encoding Mth373 from *M. thermautotrophicus* ΔH was cloned the same as that for MTBMA1234 [26], and the protein expressed and purified as previously [26], except for the following changes. The primers used were as follows: forward 5′‐CACCATGTTACCAGGAAAACCCTTATTC and reverse 5′‐TCAGTCCTCTATCCTGTAGTACC. Recombinant hexahistidine‐tagged Mth373 was expressed from *E. coli* LOBSTR BL21* (Kerafast, Newark, CA, USA) grown initially at 37 °C with the temperature lowered after induction to 22 °C for 16 h. After nickel‐affinity purification, the buffer was exchanged by dialysis to 20 mm MOPS pH 7.0 containing 2 mm tris(2‐carboxyethyl)phosphine (TCEP) and 500 mm KCl.

### Crystallisation and structure determination of Mth375

Mth375 (designated Mth375_AU; 10 mg/mL in 20 mm MOPS pH 7.0, 5% (v/v) glycerol, 25 mm KCl, 7 mm β‐mercaptoethanol and 1 mm DTT) was initially crystallised using the sitting drop method at 21 °C in a number of conditions using the Molecular Dimensions JCSG‐plus screen and Structure Screens 1 + 2 [58]. Crystallisation was optimised via the addition of 1.0 mm uridine 5′‐diphosphate (UDP) to Mth375 and for the most promising condition using the Silver Bullets Bio screen from Hampton Research. Crystals were harvested from wells with mother liquor containing 1.0 m ammonium phosphate monobasic, 0.1 m sodium citrate pH 5.6 and Silver Bullets Bio condition H9. A second solution of Mth375 (designated Mth375_NU) was prepared with 1.0 mm UDP for co‐crystallisation. Crystals were obtained at 21 °C utilising the ShotGun screen from Molecular Dimensions [59] and optimised using the additive screen from Hampton Research with a resulting final mother liquor of 0.1 m NaCl, 1.6 m (NH_4_)_2_SO_4_, 0.1 m sodium HEPES pH 7.5 and 3% (w/v) 1,8‐diaminooctane. These crystals were then soaked in 10 mm *N*‐acetyl‐d‐GlcN for several minutes prior to freezing in liquid nitrogen to help identify the sugar substrate binding domain. Mth375 crystals were harvested within 1‐4 weeks of incubation at 21 °C. X‐ray diffraction data was collected at 100 K from flash‐cooled crystals in cryoprotectant containing perfluoropolyether oil for Mth375_AU or mother liquor with added 25% (v/v) ethylene glycol for Mth375_NU at the Australian Synchrotron MX beamlines [60, 61] using *Blu‐Ice* [62] and processed with *XDS* [63] and *Aimless* [64]. The exposure time, oscillation range, crystal‐detector distance and beam attenuation were adjusted to optimise the collection of data to a resolution of 2.30 and 2.01 Å, respectively. Further data collection and processing statistics are shown in Table 1. Initial phases for Mth375_AU were determined by the molecular replacement program *Phaser* [65], within the *CCP*4 program suite [66], using the crystal structure of a sugar epimerase from the Gram‐negative bacterium *Bordetella bronchiseptica* (PDB: 2PZJ) [34] as the search model. Difference‐Fourier maps (2F_o_‐F_c_ and F_o_‐F_c_) were visualised in *Coot* [67] and enabled the inclusion of additional amino acid side chains, associated molecules from the mother liquor and water molecules. Structural idealisation and restrained refinement were carried out using *REFMAC*5 [68]. Initial phases for Mth375_NU were determined by the molecular replacement program *MolRep* [69] using the refined crystal structure of Mth375_AU. Mth375_NU structural idealisation and restrained refinement were carried out in an identical manner to Mth375_AU. X‐ray diffraction and final refinement statistics are provided in Table 1. Structural coordinates and structure factors have been deposited in the RCSB Protein Data Bank (PDB) under accession codes PDB: 6PMH (Mth375_AU) and PDB: 6PNL (Mth375_NU).

### Crystallisation and structure determination of Mth373

Mth373 coenzyme bound forms (designated Mth373_NU and Mth373_NX; 4.1 mg/mL in 20 mm MOPS pH 7.0, 2 mm TCEP, 500 mm KCl and 2.0 mm NADH) were crystallised with the sitting drop method at 21 °C using the PEGRx screen from Hampton Research. Mth373_NU contained an additional 1.0 mm UDP and Mth373_NX contained 1.0 mm uridine 5′‐diphosphoglucuronic acid (UGA). Crystals were harvested from wells with mother liquor conditions 0.2 m MgCl_2_, 0.1 m sodium citrate tribasic dihydrate pH 5.0, 10% (w/v) polyethylene glycol (PEG) 20,000 (Mth373_NU) and 0.1 M sodium citrate tribasic dihydrate pH 5.5, 18% (w/v) PEG 3350 (Mth373_NX). Crystals of either complex were harvested within 1‐4 weeks of incubation at 21 °C. X‐ray diffraction data were collected at 100 K from flash‐cooled crystals in mother liquor containing 25% (v/v) ethylene glycol at the Australian Synchrotron MX beamlines using *Blu‐Ice* [62] and processed with *XDS* [63] and *Aimless* [64]. The exposure time, oscillation range, crystal‐detector distance and beam attenuation were adjusted to optimise the collection of data to a resolution of 2.00 and 1.96 Å, for Mth373_NU and Mth373_NX, respectively. Further diffraction data collection and processing statistics are shown in Table 1. Initial phases for Mth373_NU were determined by the molecular replacement pipeline *BALBES* [70] using the dTGD crystal structure from *Salmonella enterica* (PDB: 1KEW) [71], with initial automated model building and refinement using the online ARP/wARP 7.6 server [41]. Difference‐Fourier maps (2F_o_‐F_c_ and F_o_‐F_c_) were visualised in *Coot* [67] and enabled the addition of further amino acid side chains, associated molecules from the mother liquor and water molecules. Further structural idealisation and restrained refinement was carried out using *REFMAC*5 [68]. Initial phases for Mth373_NX were determined by the molecular replacement program *MolRep* [69] and the refined crystal structure of Mth373_NU. X‐ray diffraction data and final refinement statistics are listed in Table 1. Structural coordinates and structure factors for Mth373_NU and Mth373_NX have been deposited in the RCSB PDB under accession codes PDB: 8W3U and PDB: **9AR1**, respectively. Attempts to produce crystal structures of additional *M. thermautotrophicus* enzymes Mth1789, Mth631 and Mth380 failed to produce crystals of sufficient quality for crystal structure determination.

### Molecular modelling

Structural homologues of Mth375 were identified utilising the online structural alignment server CLICK [37]. The detection of similarities in structural subdomains enabled the deeper inspection of NS‐SDR active sites accounting for their potential induced fit plasticity when UDP‐sugar substrates are bound. The selected structures (Table 2) with varying activities or bound substrate were submitted to SALIGN [72] and the resultant structure‐based sequence alignments were visualised utilising Clustal Omega (0.1.2.4) multiple sequence alignment online server [38]. *In silico* models of *M. thermautotrophicus* enzymes Mth1789, Mth631 and Mth380 were developed using the ICM‐Homology modelling algorithm, utilising the full refinement of side chains and loops option available in the ICM‐Pro modelling suite (Molsoft LLC; www.molsoft.com) [50, 51]. ICM‐Pro was also used for template searches for our candidate proteins, allowing for automated alignment and inspection prior to modelling the target proteins, including associated cofactors and substrates where possible. Protein identities and template details are shown in Table 4. The resultant models of Mth1789, Mth631 and Mth380, and the crystal structures of Mth375_NU (PDB: 6PNL) and Mth373_NX (PDB: 9AR1) were superimposed using SALIGN [72] and aligned sequences visualised using Clustal Omega (0.1.2.4) multiple sequence alignment online server [38] (see Table 4). Molecular docking experiments were carried out on the crystal structure of Mth375 (PDB: 6PNL) using the program GOLD (Genetic Optimization for Ligand Docking) version 5.2 favouring the GOLD Scoring system [39]. The search domain was centred on the terminal phosphate of the bound UDP molecule with radius set to 15 Å and executed using a 100% search efficiency, generating 10 Genetic Algorithm (GA) runs. A scaffold‐based constraint was also employed so that docked compounds mimicked the bond overlap observed in the bound UDP molecule. All library definitions for the rotameric state of residues Asn91, Ser129, Cys131, Tyr155, Asn184, Asn197, Thr215, Arg281 and Trp283 were allowed during the docking run. The generated binding poses of potential substrates were inspected, and conformations were chosen for further analysis taking into account their ranking and interactions with active site residues. The structural context for sequence conservation was analysed using the ConSurf server [73].

### Phylogenetic analysis of hexose epimerase sequences

The epimerase amino acid sequences in a previously published phylogenetic analysis [36] together with sequences for methanogen and homologous protein crystal structures were downloaded from the nonredundant NCBI or RCSB databases and imported into MEGA7. The sequences were aligned using Clustal and a Neighbour‐Joining phylogenetic tree [74] constructed in MEGA7 using default parameter settings [75].

## Conflict of interest

The authors declare that they have no conflicts of interest with the contents of this article.

## Author contributions

RR conceptualisation; RR, LS, VC, PE and AS‐S methodology; VC data collection, curation and formal analysis; RR, VC, LS and AS‐S writing and review; VC visualisations; R.R. supervision, project administration and funding acquisition.

## Data Availability

The data supporting the findings of this study can be found within the article. Structural coordinates and structure factors can be found in the RCSB, with PDB codes 6PNL (https://www.rcsb.org/structure/6PNL), 6PMH (https://www.rcsb.org/structure/6PMH), 9AR1 (https://www.rcsb.org/structure/9AR1) and 8W3U (https://www.rcsb.org/structure/8W3U).
